# Office- vs Hospital-Based Cardiac Imaging Among Part B Medicare Beneficiaries for Evaluating Coronary Artery Disease

**DOI:** 10.1016/j.jacadv.2026.102707

**Published:** 2026-04-03

**Authors:** Ahmed Sayed, Maria Alwan, Ahmad Al Yaman, Mahmoud Al Rifai, Mouaz H. Al-Mallah

**Affiliations:** a Rochester General Hospital, Rochester, New York, USA; b Houston Methodist DeBakey Heart & Vascular Center, Houston, Texas, USA

**Keywords:** cardiac magnetic resonance, computed tomography angiography, coronary artery disease, echocardiography, positron emission tomography

**What is the clinical question being addressed?**

Where does most cardiac imaging for the assessment of coronary artery disease take place?

**What is the main finding?**

Approximately half of scans for evaluating coronary artery disease take place in hospital-based facilities, but this varies significantly by modality.

The setting in which medical imaging is performed (office-based vs hospital-based facilities) has important implications with respect to cost, availability, and reimbursement. Over the past decade, a number of changes have occurred which may impact the location of cardiac imaging, including the adoption of different imaging modalities,^1^^,^^2^ greater takeover of independent physician practices by private equity, and changing reimbursement patterns. Accordingly, we sought to evaluate the proportion of cardiac imaging procedures performed in office-based vs hospital-based settings across different modalities.

Data from 2013 to 2023 on the number of cardiac imaging procedures for coronary artery disease assessment for Medicare Part B beneficiaries was obtained from the Centers for Medicare & Medicaid Services across 5 cardiac imaging modalities: single-photon emission computed tomography (CT) (SPECT) (current procedural terminology codes 78451 and 78452), positron emission tomography (PET) (78431 and 78492), CT angiography (CTA) (75574), magnetic resonance imaging (MRI) (75563), and echocardiography (93350 and 93351).^3^ The data set classifies location as facility-based (primarily hospital departments) or non–facility-based (primarily physician offices).^4^ The number of scans performed in either setting was quantified annually from 2013 to 2023. Scans were standardized per 1 million beneficiaries to account for the changing size of the Medicare population. Because the primary unit of this analysis was the number of scans (not beneficiaries), each scan was counted separately, even if performed for the same beneficiary. In addition, the proportion of scans performed in either setting was calculated across different states. Analyses were performed using R (version 4.2.0, R Foundation for Statistical Computing). This study was exempt from Institutional Review Board approval as it does not use patient-level data. The statistical code required to replicate this analysis is publicly available and, in combination with the publicly available nature of the data set, can be used to replicate our findings.^5^

The overall proportion of cardiac imaging scans performed in office-based vs hospital-based settings was similar in 2013 (54.3% vs 45.7% respectively) and 2023 (56.0% vs 44.0% respectively). However, distinct temporal trajectories were observed for different modalities, with a significant rise in the number of office-based PET procedures (from 2,322-3,444 per 1 million beneficiaries), such that office-based PET procedures comprised 82.8% of all PET procedures in 2023 (Figure 1A). Conversely, hospital-based procedures grew more rapidly for MRI (43-143 per 1 million beneficiaries) and CTA (717-4,010 per million beneficiaries). The use of SPECT and echocardiography declined, both for hospital and office-based procedures. Significant geographic variation was observed across different U.S. states. The highest proportion of office-based imaging was in Arizona (80.1%), Maryland (79.6%), Delaware (74.7%), and New York (74.2%), and the lowest proportions in South Dakota (5.0%), North Dakota (5.7%), Maine (6.3%), and Vermont (7.0%) (Figure 1B).

**Figure 1:**
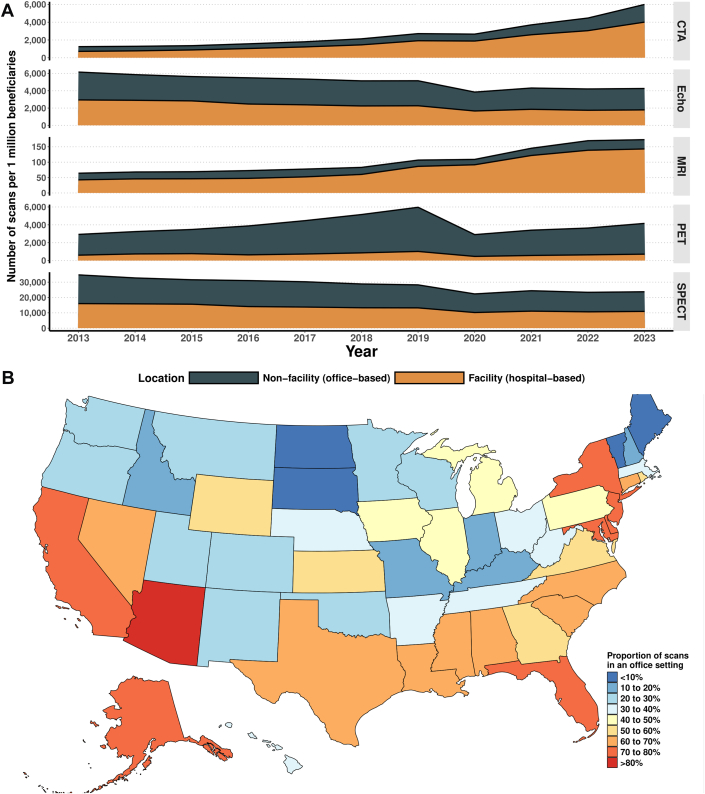
Trends in the Location of Cardiac Imaging and State-Level Variation in Location of Cardiac Imaging (A) Trends in the location of cardiac imaging, stratified by modality. On the Y-axis, the number of cardiac imaging procedures for the assessment of coronary artery disease, standardized to per 1 million part B beneficiaries to adjust for the changing size of the Medicare population, is displayed. (B) State-level variation in location of cardiac imaging in 2023. Color intensities represent the proportion of cardiac imaging (as a percentage of the total number of cardiac imaging scans in a given state) for the assessment of coronary artery disease performed in office settings for each state. CTA = computed tomography angiography; MRI = magnetic resonance imaging; PET = positron emission tomography; SPECT = single-photon emission computed tomography.

The striking increase in office-based PET imaging likely relates to several factors, although we caution that the following proposed explanations are speculative in nature. Since many offices traditionally performed SPECT, a transition to PET (as opposed to switching to a different modality) would require fewer infrastructural changes and retraining of both technical and clinical personnel, in addition to having higher reimbursement rates.

In contrast, there was a greater uptake of cardiac CTA in hospital-based facilities, which likely repurposed CT machines and already had the infrastructure and technical personnel to perform CT imaging. Similarly, cardiac MRI requires substantial upfront investment and additional retraining that would deter most offices, particularly if imaging volume is not high enough to offset upfront costs. The combination of PET’s high reimbursement rates with manageable setup expenses probably made it a more economically advantageous proposition to independent practices compared to other imaging modalities. Furthermore, hospitals may be better suited to handle lower reimbursement rates for CTA/MRI due to the use of bundled payment structures or partially subsidizing these services using other more profitable services.

State-level variation likely represents a confluence of different factors, although we acknowledge that concrete data quantifying the contribution of each are lacking. A number of U.S. states have “*Certificate-of-Need”* laws wherein obtaining regulatory approval is a necessary pre-requisite to the acquisition of advanced imaging equipment. These laws likely disproportionately affect independent physician offices because the finances required to obtain regulatory approval are substantial. In addition, some states have more prior authorization requirements for imaging performed in an outpatient vs inpatient setting. Furthermore, different states reimburse independent physician-owned offices at different rates to hospital-based facilities for the same imaging procedure. Accordingly, it is unsurprising that some states with policies more financially favorable to physician-owned imaging facilities have a proportion of office-based cardiac imaging that is greater than the national average. Lastly, takeovers of independent physician offices by larger hospital-based systems have also likely affected the availability of office-based imaging procedures. This availability is important for 2 primary reasons. First, office-based imaging is generally less costly to the health care system than hospital-based imaging. Second, a greater availability of office-based imaging procedures allows a greater number of patients to access these services and reduces waiting times.

This analysis’ primarily limitation is the inability to assess the costs of office-based vs hospital-based imaging, primarily because facility fees for hospital-based imaging were not included in the data set. Because imaging performed in hospital-based facilities has higher reimbursement rates, prior analyses have estimated the excess annual cost of performing cardiac imaging in hospital-based facilities at $500 million for CMS and $161 million for patients.^6^ Second, as mentioned previously, the proportion of imaging performed in physician-owned offices is affected by multiple factors, and it is difficult to distinguish the largest contributors to observed state-level variation using this data. Third, this analysis is limited to Medicare beneficiaries and its findings may not generalize to other individuals. Younger patients may have different indications for cardiac imaging (eg, diagnosing cardiomyopathies using MRI rather than diagnosing coronary artery disease using CTA/nuclear imaging). Future analyses with access to data from private insurers would be helpful. Fourth, classifying imaging as being facility vs non–facility-based may overlook important nuances (eg, hospital-affiliated outpatient facilities billing under nonfacility rates).

## Funding support and author disclosures

Dr Al-Mallah has received research support from 10.13039/100004340Siemens and 10.13039/100004313General Electric, unrelated to this work; and he is also a consultant to 10.13039/100004313General Electric, 10.13039/100004319Pfizer, Medtrace and Jubilant. All other authors have reported that they have no relationships relevant to the contents of this paper to disclose.
